# Probing the Dynamic Structural Evolution of End-Functionalized Polybutadiene/Organo-Clay Nanocomposite Gels before and after Yielding by Nonlinear Rheology and ^1^H Double-Quantum NMR

**DOI:** 10.3390/polym14081518

**Published:** 2022-04-08

**Authors:** Wansu Peng, Chengdong Feng, Jiawen Hou, Rongchun Zhang, Pingchuan Sun, Yun Gao, Xiaoliang Wang

**Affiliations:** 1 Key Laboratory of High Performance Polymer Materials and Technology of Ministry of Education, Department of Polymer Science and Engineering, School of Chemistry and Chemical Engineering, Nanjing University, Nanjing 210023, China; mg1924065@smail.nju.edu.cn (W.P.); mg1924018@smail.nju.edu.cn (C.F.); p709@nju.edu.cn (Y.G.); 2 Key Laboratory of Functional Polymer Materials of Ministry of Education and College of Chemistry and School of Physics, Nankai University, Tianjin 300071, China; 2120200839@mail.nankai.edu.cn (J.H.); spclbh@nankai.edu.cn (P.S.); 3 South China Advanced Institute for Soft Matter Science and Technology (AISMST), School of Emergent Soft Matter, Guangdong Provincial Key Laboratory of Functional and Intelligent Hybrid Materials and Devices, South China University of Technology, Guangzhou 510640, China; zhangcr@scut.edu.cn

**Keywords:** nanocomposite gel, dynamics structure evolution, yielding, solid-state NMR, rheology

## Abstract

Understanding the structural evolution process after the yielding of networks in polymer nanocomposites can provide significant insights into the design and fabrication of high-performance nanocomposites. In this work, using hydroxyl-terminated 1,4-polybutadiene (HTPB)/organo-clay nanocomposite gel as a model, we explored the yielding and recovery process of a polymer network. Linear rheology results revealed the formation of a nanocomposite gel with a house-of-cards structure due to the fully exfoliated 6 to 8 wt% organo-clays. Within this range, nonlinear rheologic experiments were introduced to yield the gel network, and the corresponding recovery processes were monitored. It was found that the main driving force of network reconstruction was the polymer–clay interaction, and the rotation of clay sheets played an important role in arousing stress overshoots. By proton double-quantum (^1^H DQ) NMR spectroscopy, residual dipolar coupling and its distribution contributed by HTPB segments anchored on clay sheets were extracted to unveil the physical network information. During the yielding process of a house-of-cards network, e.g., 8 wt% organo-clay, nearly one-fourth of physical cross-linking was broken. Based on the rheology and ^1^H DQ NMR results, a tentative model was proposed to illustrate the yielding and recovery of the network in HTPB/organo-clay nanocomposite gel.

## 1. Introduction

A polymer matrix constructed with nanoparticle networks inside is usually an efficient strategy to achieve a high-performance material in industry [1,2,3,4]. Yielding the inner nanoparticle networks, especially nanoparticles with large aspect ratios, would lead to huge differences in structures, properties, and end uses in comparison to materials with intact networks [5]. Clay is a kind of typical nanoparticle with a larger aspect ratio, and well-exfoliated clay sheets may construct a house-of-cards structure in the polymer/clay nanocomposites [6,7,8]. Different degrees of yielding may induce different inner networks, resulting in versatile material performance [9]. Thus, understanding and controlling the yielding and aligning processes are considered to be very important in polymer science and engineering.

The linear and nonlinear viscoelastic behavior of complex fluids in nanocomposites reflects information on the microstructure and molecular interactions therein [10,11,12,13,14,15,16,17]. Small-amplitude oscillatory shear (SAOS) experiments were the canonical method for probing the linear viscoelastic properties of nanocomposites with intact structures, while large-amplitude oscillatory shear (LAOS) experiments or startup flow were used to yield the inner structure and show information thereafter, which controlled the system response in most processing operations [9,18,19]. Wu et al. reported that the transient stress overshoot behaviors in polylactic acid/clay and polylactic acid/graphene nanoplatelets were tightly linked with the percolation network density [20,21,22]. Winter et al. used the liquid rubber/clay nanocomposites as a model sample to elucidate the yielding process by applying LAOS experiments [23,24]. Solomon et al. reported that the attractive interactions between the multi-nanosheet domains in polypropylene/clay would lead to the quiescent structure evolution and network reconstruction after shearing [3]. Their research showed that the rheological measurement of polymer/clay nanocomposites could reveal the generation, destruction, and reconstruction of the network, and the responses of the highly anisotropic layers dispersed in the viscoelastic polymer matrix. Until now, a molecular-level understanding of structural changes before and after yielding was still missing.

Solid-state nuclear magnetic resonance (NMR) technology has developed rapidly in the past few decades and has gradually become an irreplaceable tool for characterizing polymer microstructures and segment dynamics at the molecular level [25,26,27]. In polymer-based nanocomposites or gel, due to the existence of cross-linking points, the chain segments between cross-linkages typically undergo anisotropic motions, resulting in a residual dipolar coupling (*D*_res_) among protons, typically around kHz [28]. As a result, *D*_res_ and its distribution were a unique prober, as extracted from proton double-quantum (DQ) NMR experiments, to explore the cross-linking density [29,30,31] and structural heterogeneity [32,33] of the network. Zhang et al. used DQ NMR to explore the hydrogen-bonding network of self-healing materials [29]. Kay et al. used it to reveal the contribution of grafted silica to the cross-link density of the elastomer matrix [34]. Wu et.al. showed the inhomogeneity in the network of natural rubber via ^1^H DQ NMR [35]. However, few works on yielding a nanocomposite by ^1^H DQ NMR have been conducted yet, partly because of the lack of a model sample with a well-defined network inside that could be yielded within the NMR experiment temperature range.

In our previous works, we found that highly anisotropic, organically modified silicates (organo-clays) exfoliated when embedded in dicarboxyl-terminated 1,4-polybutadiene (CTPB) or hydroxyl-terminated 1,4-polybutadiene (HTPB), up to a clay concentration of 10 wt% [36,37,38]. After having mixed the clay into the polymer, rheological experiments identified the exfoliation process at different temperatures [37,39]. Such exfoliated organo-clay nanosheets could form an ideal structure of a “house-of-cards”, which could serve as a reference state to monitor the structural ripening process [40]. A regular rheological pattern therein was found, which might be typical for physical gelation [40], and the equilibrium segmental dynamics therein were identified by solid-state NMR [41,42]. The temperature dependence of structural development in the nanocomposite had been studied utilizing time-resolved mechanical spectroscopy (TRMS) [43]. Nevertheless, until now, a detailed understanding of the yielding process and dynamic evolution thereafter at a molecular level in a polymer/clay nanocomposite physical gel remained an open challenge [44,45].

The current study aims to understand the yielding process and the following structural evolution of polymer/clay nanocomposite gels. HTPB/organo-clay nanocomposite served as a model sample where the clay sheets could be well exfoliated. The complete evolution process from sol to gel, and then the loose to dense network transition, was revealed by SAOS. Then, startup shear experiments were used to quantitively break the equilibrium three-dimensional network, followed by exploring the movement of organo-clay nanosheets. Furthermore, network structures under different yielding/relaxation processes were quantitatively revealed by ^1^H DQ NMR. On the basis of rheology and ^1^H DQ NMR results, a tentative model was proposed to elucidate the evolution of the structure and segmental dynamics in the HTPB/C18-clay nanocomposite gel.

## 2. Materials and Methods

### 2.1. Materials

Hydroxyl-terminated 1,4-polybutadiene oligomers (HTPB28) of *M*_n_ = 2800 g/mol and carboxyl-terminated 1,4-polybutadiene oligomers (CTPB) of *M*_n_ = 4200 g/mol were purchased from Aldrich Chemical Co., St. Louis, Missouri, United States. The organoclay containing octadecyltrimethylammonium chloride (C18A), denoted as C18-clay, was purchased from Fenghong New Material Co. Ltd., Huzhou, China.

### 2.2. Sample Preparation

HTPB28/C18-clay pre-nanocomposite gel could be conveniently prepared by gently and quickly mixing the HTPB28 with organo-clay at room temperature [46,47]. In order to obtain samples with intact network structures, they were loaded onto the fixture for in situ ripening (heated from 25 °C to 120 °C with 2 °C/min and rested for 5 min at 120 °C). Through this method, a well-developed structure could serve as a repeatable initial state for the following rheological and NMR measurements [36,40]. The exfoliation of organo-clay sheets was further proved by TEM images (see Supplementary Materials, Figure S1) and XRD results (see Supplementary Materials, Figure S2).

### 2.3. Rheology Experiment

Rheology experiments were all carried out on a strain-controlled rheometer ARES-G2 (TA Instruments, New Castle, Delaware, United States). A 25 mm diameter parallel plate with a gap between 0.7 and 1.0 mm was used in linear viscoelastic measurements. To protect samples from degradation, experiments above 80 °C were all carried out under a nitrogen atmosphere. Small amplitude oscillatory shear (SAOS) from 100 to 0.1 rad/s was performed in a temperature range from −60 to 20 °C (0–8 wt%) and 0 to 120 °C (5–16 wt%) with an interval of 20 °C in the linear region (e.g., 1% strain rate).

The startup of steady shear and flow reversal measurements was conducted by using a set of 8 mm cone and plate geometry with a 0.1 rad cone angle. The experimental procedures of flow reversal measurements were as follows: Firstly, a conventional startup of steady shear with a 10 s^−1^ shear rate was applied. Then, the flow was stopped and the sample was given a certain time to recover. At last, a shear flow with the same shear rate but opposite direction to the initial startup experiment was applied.

### 2.4. Proton Double-Quantum (DQ) NMR Experiment

All the proton DQ NMR experiments were carried out on a Bruker Minispec (Billerica, Massachusetts, United States) mq20 at a proton resonance frequency of 20 MHz. The sample temperature was controlled by a BVT3000 heater (Bruker Minispec, Billerica, Massachusetts, United States) with an accuracy of ±0.1 °C. The 90° pulse length is about 3.0 μs, and the receiver dead time was about 13 μs. A homemade Teflon coaxial cylinder, see Supplementary Materials Figure S8a, was put into the 10 mm NMR tube, which could be used to yield the sample as we did in the rheometer. Unannealed samples were put into the NMR tube with a sample height of around 10 mm. The ripening process was carried out in situ by heating up from 25 °C to 120 °C, holding for 5 min at 120 °C, then cooling down to room temperature. MAPE-DQ experiments [48] were performed for all the samples, where a MAPE (magic and polarization echo) [49] dipolar filter was implemented right before the DQ recoupling sequences (i.e., Baum–Pine pulse sequence [50]) in order to eliminate the interference of the signals from a modifier (C18A) anchored on the nanosheets. The total MAPE filter time was set as 0.32 ms in the MAPE-DQ experiments. The detailed principle of DQ NMR, as well as the data processing procedures, can be found in the Supplementary Materials.

## 3. Results

### 3.1. Structural Evolution of Nanocomposite Gels in the Linear Region

**Figure 1:**
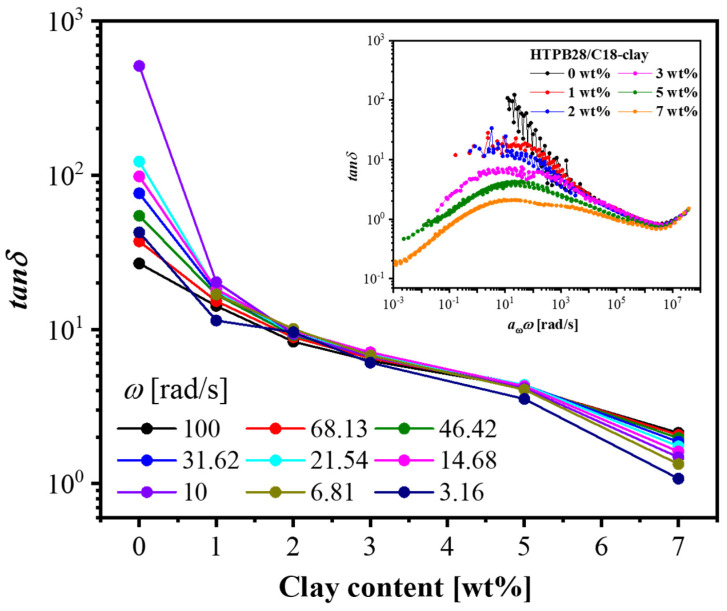
Plots of loss factor *tan* δ in a frequency range from 100 rad/s to 1 rad/s (black to purple) as a function of clay content (conducted at 20 °C). (Inset) *tan* δ as a function of angular frequency (*T*_ref_ was 20 °C).

SAOS was conducted to explore the gradual formation of network structures and the diffusion process of nanoparticles in polymer nanocomposites. Master curves obtained by time-temperature superposition (TTS) are shown in Figures S3 and S4 and the shift factors belonging to the SAOS master curves are shown in Figure S5. When the concentration of C18-clay was lower than or equal to 2 wt%, plateau moduli did not exist in the low-frequency region, displaying a terminal relaxation behavior. As the concentration of C18-clay increased to 3 wt%, the modulus plateau formed. Plotting *tan* δ under different frequencies as a function of C18-clay content [51] gave a more intuitionistic sol-to-gel transition process (see Figure 1). According to the clear frequency-invariant crossover of curves, we could identify that a sol-to-gel transition occurred during the range of 2–3 wt%, whereas the physical gel only had a loose network at this time, inferred from the weak plateau moduli in Figure S3a. For the sake of showing explicit nonterminal behaviors at low concentrations, *tan* δ was superposed onto the bulk HTPB28 (see the inset of Figure 1). In the high-frequency region, we inferred from the superior overlap of loss factor that the relaxation behavior was still dominated by polymer, despite the hydrodynamic contribution of nanosheets. Therefore, we can refer to this region as the polymer-dominated hydrodynamic part [52]. In the low-frequency terminal relaxation region, *tan* δ curves showed a significant upward trend as the polymer concentration decreased, suggesting that the terminal relaxation was mainly controlled by physical interactions in the polymer/organo-clay network.

**Figure 2:**
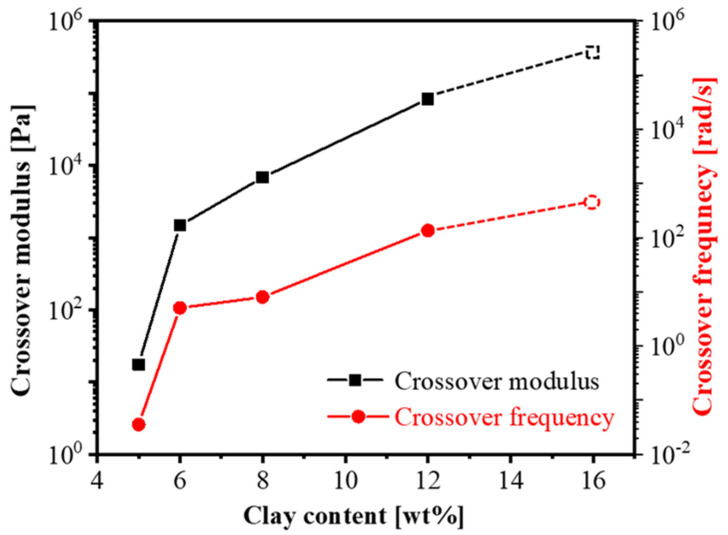
Crossover modulus and frequencies of HTPB28/C18-clay (5–16 wt%) nanocomposites in TTS. The sample with a 16 wt% concentration does not have a clear intersection point within the test temperature range, so the maximal values of storage modulus and frequency obtained from the experiment are used as substitutes and displayed in the form of dotted points.

The master curves of the nanocomposite gel with high clay concentration (5–16 wt%), as shown in Supplementary Materials Figure S4a, changed significantly when compared with low-concentration ones. The shift factors belonging to the SAOS master curves are shown in Figure S5b. Corresponding crossover modules and frequencies increased with increasing clay concentration, owing to enhanced restrictions of the organic–inorganic interface, as shown in Figure 2. Such results were in good agreement with our previous studies [41]. Furthermore, the crossover modulus and frequency augmented two orders of magnitude from 5 wt% to 6 wt%. Such a surge indicated the formation of a dense network structure with direct contact between nanosheets, forming a house-of-cards structure [13]. As a comparison, the modulus curve in polybutadiene/C18-clay 12 wt% did not have an explicit plateau modulus except at a very low frequency since the polybutadiene chains without end-functional groups were difficult to get access to the clay surface, leading to an absence of a network structure (see Figure S4b in the Supplementary Materials).

**Figure 3:**
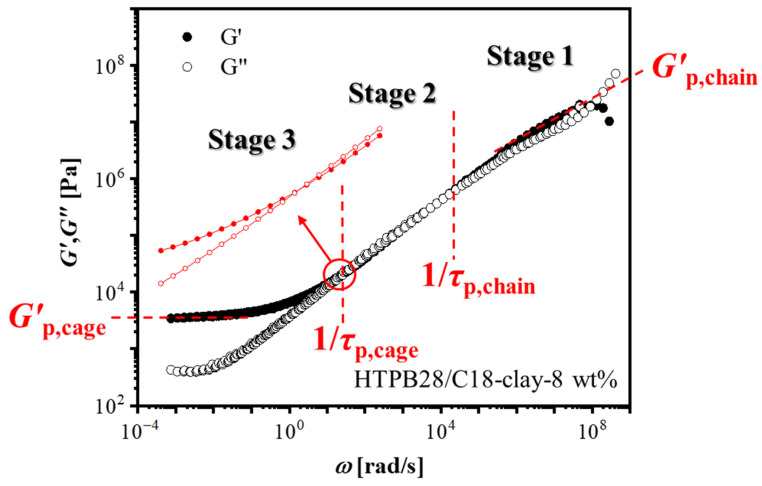
Master curve of HTPB28/C18-clay with 8 wt% clay constructed from the SAOS curves at temperatures between −60 to 120 °C with 20 °C intervals. The reference temperature (*T*_ref_) = 20 °C. Solid symbols represent the storage modulus and hollow symbols represent the loss modulus.

From the results of Figure 1 and Figure 2, we can define a sol-to-gel (2–3 wt%) and loose-to-dense (5–6 wt%) network transition at a very low percolation concentration of around 5 wt%. Furthermore, the diffusion process of nanosheets in a polymer matrix could be revealed by the linear rheology, e.g., HTPB28/C18-clay 8 wt%, shown in Figure 3. According to the diffusion dynamics model of nanoparticles in a polymer matrix described by You et al. [53] the master curve can actually be split into three stages:

In the first stage, nanosheets are confined in a network of end-functionalized polymers [54], so the relaxation process is delayed corresponding to the first stage modulus, *G*′_p,chain_. The shape of *G*′_p,chain_ is nearly identical to the *G*′_bulk_ of bulk HTPB28 in the high-frequency region (see Figure S3a), implying that the relaxation mode of the nanocomposite in a short time scale is similar to bulk polymer. However, the corresponding modulus of HTPB28/C18-clay would be higher, which is mainly due to the hydrodynamic interactions between HTPB28 and C18-clay [52,55].

In the second stage, the dynamic modulus starts to go down, indicating that the nanosheets begin to escape from the end-functionalized polymer network. Owing to the existence of interactions between polymers and nanosheets, the diffusion of nanosheets is actually still limited.

In the third stage, after escaping from the end-functionalized chain network, the target nanosheet will be trapped in a new round of constraints caused by the surrounding nanosheets, thereby forming the second plateau modulus, *G*′_p,cage_.

From the results of small-amplitude oscillatory shear, it was found that the diffusion of nanosheets was similar to spherical nanoparticles, e.g., 30 wt% [53], yet the critical concentration (percolation threshold) forming a network structure was greatly reduced in nanocomposites with nanosheets, e.g., 6 wt% in HTPB28/C18-clay.

### 3.2. Fracture of Networks by Transient Nonlinear Rheology

Rheological experiments in the linear region provide effective information about the microstructure of nanocomposites [6,11,14,56]. However, it was only valid when the total deformation was quite small, which hindered us from exploring the properties of the network and the motion of nanosheets. Herein, startup shear experiments were implemented to probe transient rheological behaviors of nanocomposites.

**Figure 4:**
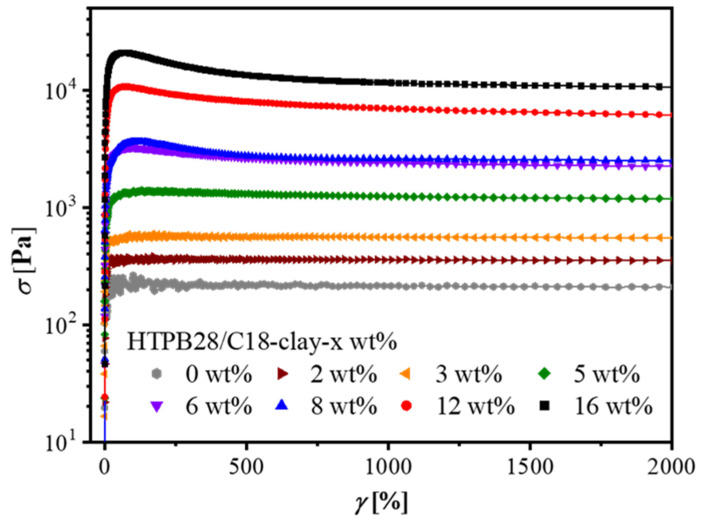
Concentration dependence of HTPB28/C18-clay at a startup shear at 20 °C. A shear rate of 10 s^−1^ is big enough to break the network structure.

We applied a startup shear with a shear rate of 10 s^−1^ to nanocomposites with different concentrations (see Figure 4). Based on the linear rheology results, we selected several representative concentrations which could perfectly reveal the key changes during structural evolution. Transient stress overshoots were dominated by nanosheet contents. The more nanosheet content added, the higher stress overshoots became, which was due to a denser network structure when the filler concentration was beyond 5 wt%. It is worth noting that a nanocomposite containing 2 wt% clay did not show any stress overshoot behavior. When clay loading up to 3 wt%, the stress overshoot then appeared. Another special phenomenon occurred between 5 wt% and 6 wt%; an obvious change from a weak stress overshoot to a strong stress overshoot. These two distinct results were not surprising because they were in perfect agreement with the results in the linear region. Now, we have obtained two separate pieces of rheological evidence proving the sol-to-gel transition in 2–3 wt%, and the loose-to-dense network transition in 5–6 wt%.

Note that in Figure 4, all stress overshoots occurred under the same *γ* of about 100%; the same results were found in a shear rate dependence experiment (see Figure S6 in the Supplementary Materials). Before the critical strain, only elastic deformation took place, so the network remained intact. After the critical strain, deformation was no longer recoverable due to the destruction of the network. It required a long process for the stress curve to flatten out. In other words, the reconstruction of the polymer network obeyed a slow relaxation mode [57], ascribed to the rotation or roll-over relaxation of clay nanosheets under ongoing shear.

### 3.3. Recovery Properties of the Network

**Figure 5:**
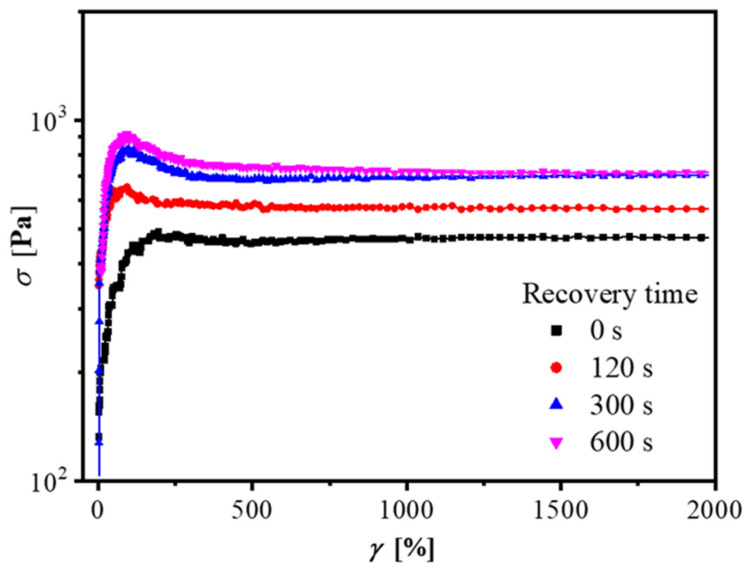
Transient stress response for HTPB28/C18-clay 8 wt% with different recovery times in the reverse flow at 60 °C. A shear rate of 10 s^−1^ was chosen.

In addition to one-way startup shear experiments, flow reversal measurements were conducted to probe the recovery ability of the dynamic network formed by pairwise interactions between polar groups [36]. For the following research on the recovery ability of the dynamic network and motion pattern of nanosheets, we had to select a suitable clay concentration so as to comprise all the information needed. The selected concentration cannot be too low or too high, otherwise, we would obtain an incompact structure or the coexistence of exfoliation and an intercalation structure [36,37,39]. Hence, a concentration of 8 wt% was chosen in the following flow reversal measurements, forming a dense network with exfoliated silicate layers. At room temperature, the structure was irrecoverable for hours (see Figure S7 in the Supplementary Materials) [23]. We chose 60 °C as the experimental temperature because higher temperatures can enhance chain relaxation and thus speed up the network reconstruction process. Obviously, the magnitudes of the stress overshoots in the flow reversal measurement have a strong dependence on the rest time (see Figure 5). As the recovery time became longer, the magnitudes of stress overshoots increased. It is worth noting that the process of reconstruction was not at a constant rate, but fast in the range of 0–300 s then slow from 300 to 600 s, obeying a slow relaxation mode, as mentioned above. Through the flow reversal measurements, a complete process of structural evolution can be revealed. During the first startup shear, highly anisotropic clay sheets were oriented under the shear field due to the fracture of the percolation network [58,59]. However, the broken network will reconstruct under static conditions. Nevertheless, what was the driving force in the reconstruction process? According to previous work by Solomon and Krishnamoorti [3,60], it was sure that the Brownian motion was not the major driving force for the reorganization of the network. Assuming that the shape of the clay nanosheets dispersed in the polymer matrix was circular and plate-like, the rotational relaxation time, *t*_D_, due to Brownian motion, is approximately [16]:

(1) $$t_{D}\approx \frac{{\left(\frac{\pi }{2}\right)}^{2}}{D_{r0}}=\frac{\pi^{2}\eta_{m}d^{3}}{3k_{B}T}$$

where *D*_r0_ is the rotary diffusivity by Brownian motion, *η*_m_ is the viscosity of the polymer matrix, *d* is the diameter of the nanosheets, *k*_B_ is the Boltzmann constant, and *T* is the temperature. For HTPB28/C18-clay nanocomposites, *η*_m_ of the HTPB28 matrix at 60 °C was determined to be 2.35 Pa·s, and *d* was about 200 nm. As a result, *t*_D_ was calculated to be 6.72 × 10^7^ s, which was five orders of magnitude larger than the time scale of recovery time during the flow reversal measurements. Therefore, it can be well concluded that the Brownian motion was not the driving force for the reconstruction of the network. Instead, we argue that attractive interparticle and polymer–particle interactions are more likely to be the major driving force to promote the reconstruction of the network, as discussed below.

It should be mentioned that stress overshoots were mainly caused by fractures of the network structure, but also affected by hydrodynamic interactions and the behavior of orientations. In the normal startup experiments, the network broke down and the nanosheets oriented under the shear field, so overshoots were obviously observed, as shown in Figure 4.

**Figure 6:**
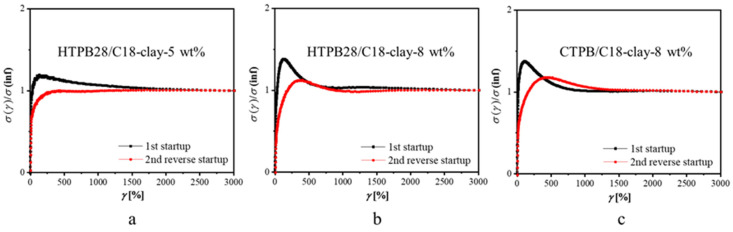
Transient stress response of samples with different clay concentrations (**a**) HTPB28/C18-Clay-5 wt%; (**b**) HTPB28/C18-Clay-8 wt% and matrix viscosity (**c**) CTPB/C18-Clay-8 wt% in the reverse flow measurements at 20 °C. A shear rate of 10 s^−1^ was chosen.

Consider the reason why stress overshoots might occur in the reverse flow without any recovery time, as shown in Figure 6. Firstly, the network structure was not completely destroyed in the forward flow. In fact, it was highly unlikely, because the applied shear rate of 10 s^−1^ was far beyond the linear response. The reverse flow was in the opposite direction against the initial shear, so it caused the rotation or roll-over of nanosheets and further led to collisions and friction between nanosheets. By increasing the clay concentration or matrix viscosity to raise the probability of collision or the difficulty of rotation, such speculation can be confirmed by the results in Figure 6. Firstly, with a higher clay concentration, more obvious stress overshoots in the reverse flow curves appeared (Figure 6a,b). This suggests that the collisions between nanosheets did affect the stress overshoots. In addition, if we replaced HTPB28 with CTPB, as shown in Figure 6b,c, a more obvious stress overshoot appeared. In our previous work, we proved that CTPB was also a good one to interact with C18-clay [37]. In the meantime, the viscosity of CTPB is 99.96 Pa·s at 20 °C, which is 3.88 times the value of HTPB28 (25.75 Pa·s at 20 °C). A higher matrix viscosity meant more difficulty in rotation, resulting in more obvious stress overshoot in the reverse flow curves. Therefore, it is reasonable that the transient overshoots were caused by the rotation or roll-over of nanosheets under reverse flow.

### 3.4. Heterogeneous Structures of Polymer-Based Nanocomposite

**Figure 7:**
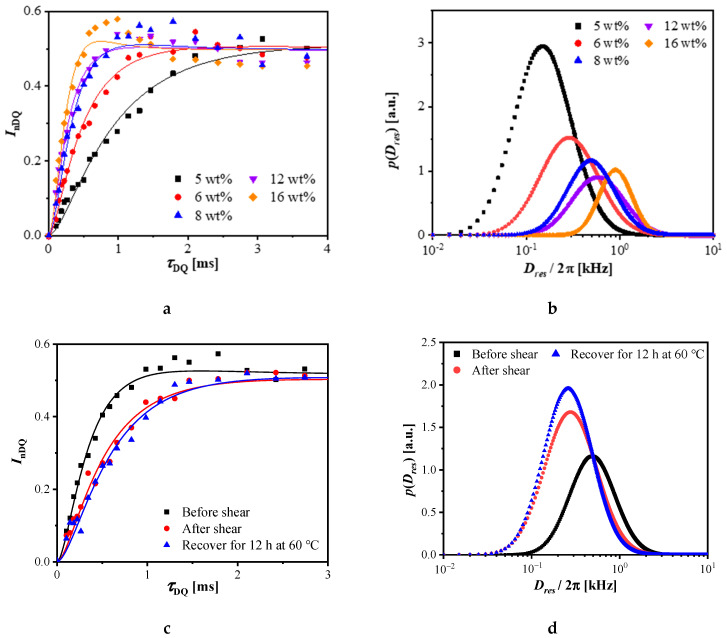
(**a**) Normalized DQ (nDQ) signal intensity as a function of DQ excitation time for the HTPB28/C18-clay nanocomposites with different clay concentrations (the solid lines are numeric fitting results via Equation (6) in the Supplementary Materials); (**b**) *D*_res_ distribution curves obtained from the numeric fitting on the nDQ curves in (**a**); (**c**) nDQ signal intensity as a function of DQ excitation time for the HTPB28/C18-clay-8 wt% nanocomposite in different states—initial state (black), after shear (red), and recovery at 60 °C for 12h after shear (blue) (the solid lines are numeric fitting results via Equation (6) in the Supplementary Materials); (**d**) *D*_res_ distribution curves obtained from the numeric fitting on the nDQ curves in (**c**). All experiments were performed at 30 °C.

Proton DQ NMR experiment is a powerful tool to probe polymer structures, such as physical cross-linking in physical gels [26]. From the DQ experiments, we could obtain two sets of data: *I*_DQ_ and *I*_ref_, which are DQ and reference signal intensity, respectively. The buildup curve of *I*_DQ_ along with *τ*_DQ_, as shown in Figure S9, suggests that the DQ signal intensity was proportional to the clay concentration due to the confinement effect of clays. Via the numerical fitting of the nDQ buildup curve, the distribution of *D*_res_ can be extracted, indicating the structural heterogeneity of the polymer network. The detail of the fitting method can be found in the Supplementary Materials and our previous works [29]. We plotted the nDQ buildup curves with different clay concentrations in Figure 7a. The nDQ signal buildup rate was proportional to the clay concentration because of the increased physical cross-linking density. There was an obvious buildup rate change between 5 wt% and 6 wt%, indicating the loose-to-dense network transition. The obtained *D*_res_ distribution curves are shown in Figure 7b, while the obtained *D*_m_ and *σ* values are summarized in Table 1. Note that in the numerical fitting of the nDQ curves, a log-normal distribution for *D*_res_ was assumed, where *D*_m_ and *σ* represent the median *D*_res_ and standard deviation, respectively. When the clay concentration increased from 5 wt% to 16 wt%, the *D*_m_ increased at the same time, indicating the enhanced constraint on polymer dynamics. However, when the clay concentration increased from 5 wt% to 16 wt%, the *σ* value decreased first, leveled off from 8 to 12 wt%, then decreased again at 16 wt%. When the clay concentration was less than 6 wt%, the exfoliated clay sheets could not reach each other to form a house-of-cards structure, resulting in a heterogeneous distribution of physical cross-linking in the gel. Therefore, the *σ* was as large as 0.71. When clay sheets were enough to form a house-of-cards structure, e.g., 8 wt%, the *σ* leveled off at a value around 0.59. However, when more clay was added, the intercalation structure dominated and the *σ* decreased again. Such structural change agrees well with the TEM and XRD results, shown in Figures S1 and S2.

As shown in the rheology sections, the physical network of HTPB28/C18-clay gels was yielded after shearing. So, we performed DQ experiments before and after shearing, as shown in Figure 7c. To perform a proper shearing, we made a Teflon coaxial cylinder in a 10 mm NMR tube, as shown in Figure S8a in the Supplementary Materials. After the MAPE-DQ experiment on the initial annealed sample, we manually applied shearing for 5 min, where the shearing was large enough to yield the physical network (network structure after manual shearing changed no more than shown in Figure S10). Then, we performed the same MAPE-DQ experiment on the sample. To explore the recovery behavior, we heated the sample to 60 °C in situ and kept it warm for 12 h, then performed the same MAPE-DQ experiment again. The nDQ signal buildup rate after shearing decreased significantly, indicating the fracture of the network, as shown in Figure 7c. There was no obvious change in the nDQ curve after recovery. That is because only applying high-temperature conditions will just induce a very weak restoration effect. However, the transient rheology experiment was very sensitive to the subtle structural changes, as shown in Figure 5, on account of the dynamic shearing field in the test. The extracted *D*_res_ distribution curves via the numerical fitting of the nDQ curves (Figure 7c) are shown in Figure 7d, while the obtained *D*_m_ and *σ* values are summarized in Table 2. After shearing, the *D*_m_ value decreased significantly from 0.69 to 0.44 kHz, which agreed well with the decreasing of modules after shearing. When enough time and temperature were given for recovering, there was little change for *D*_m_, implying that the nanosheets were oriented and part of the polymer–organo-clay interaction was thoroughly destroyed. Interestingly, yielding a well-exfoliated house-of-cards structure resulted in larger *σ* values, such as 0.68, suggesting a broader *D*_res_ distribution and a more heterogeneous network. Further recovery made the *σ* value decrease to 0.64, suggesting a less heterogeneous network thereafter.

According to the yielding results of DQ NMR, it was worth noting that the *D*_m_/2π of HTPB28/C18-clay 8 wt% reduced from 0.69 kHz to 0.48 kHz, which is close to the value of HTPB28/C18-clay 6 wt% with an intact network. When enough time and temperature were given for recovering, there was little change in *D*_m_, implying that part of the physical crosslink was thoroughly destroyed. In the HTPB28/C18-clay nanocomposite gel, the only origin of the physical crosslinks was the HTPB28 attached to the C18-clay. Thus, we could roughly estimate that 1/4 of HTPB28 segments were detached from the organo-clay surface. Interestingly, the absolute strength of the stress overshoot in reverse flow reappeared, yet was lower than the first shearing of the pristine network, as shown in Figure 6. This agreed well with the results from ^1^H DQ NMR: the interaction between HTPB28 and C18-clay lowered because of the detaching of HTPB28 from the C18-clay after yielding. However, there were still enough HTPB28 chains remaining on the C18-clay. Therefore, the C18-clay was kept well-exfoliated inside HTPB28. By the rotation or roll-over of nanosheets under reverse flow, the overshoot with lower stress remained in the reverse flow.

## 4. Conclusions

In this work, using the hydroxyl-terminated 1,4-polybutadiene (HTPB)/organo-clay nanocomposite gel as a model with a well-established network, we explored the yielding and recovering process of its network. A sol-to-gel transition occurs during the concentration range of 2–3 wt%, and the ripening process from a loose to a dense network occurred during the concentration range of 5–8 wt%. The diffusion process of 2D nanosheets was similar to the spherical nanoparticles in polymer-based nanocomposites, while the corresponding percolation threshold at which the network formed was much lower. Nonlinear rheologic experiments were introduced to yield the physical gel network, and the corresponding recovery processes were monitored. It was found that the rotation or roll-over of nanosheets under reverse flow could also induce transient overshoots. By proton double-quantum (^1^H DQ) NMR spectroscopy, residual dipolar coupling and its distribution contributed by HTPB segments anchored on clay sheets were extracted to unveil the physical network information. As the clay concentration increased, a more homogeneous network was obtained at first due to the formation of a house-of-cards structure, but then became heterogeneous owing to the dominance of the intercalation structure. During the yielding process of a house-of-cards network, e.g., 8 wt% organo-clay, nearly one-fourth of physical cross-linking was broken. Based on the rheology and ^1^H DQ NMR results, a tentative model was proposed to illustrate the yielding and recovery of the network in HTPB/organo-clay nanocomposites gel.

## Figures and Tables

**Table 1:** *D*_m_ and *σ* obtained from the fitting of the corresponding nDQ buildup curves of HTPB28/C18-clay nanocomposites with different clay concentrations by assuming a log-normal distribution for *D*_res_.

Clay Concentration	5 wt%	6 wt%	8 wt%	12 wt%	16 wt%
*D*_m_/2π (kHz)	0.25	0.48	0.69	0.86	1.06
*σ*	0.71	0.71	0.59	0.62	0.40

**Table 2:** *D*_m_ and *σ* obtained from the fitting of the corresponding nDQ buildup curves of HTPB28/C18-clay nanocomposites in different states by assuming a log-normal distribution for *D*_res_.

Status	Before Shear	After Shear	Recover
*D*_m_/2π (kHz)	0.69	0.44	0.39
*σ*	0.59	0.68	0.64

## Data Availability

The data presented in this study are available upon request from the corresponding author.

## Supplementary Materials

The following supporting information can be downloaded at: https://www.mdpi.com/article/10.3390/polym14081518/s1, Figure S1: TEM images of the (a) 8 wt% and (b) 16 wt% HTPB28/C18-clay nanocomposites after annealing above 120 ℃; Figure S2: XRD patterns of the C18-clay, PB/C18-clay-8 wt%, HTPB28/C18-clay-8 wt%, HTPB28/C18-clay-12 wt% and HTPB28/C18-clay-16 wt%; Figure S3: Master curves of (a) G′ and (b) G″ of low-concentration HTPB28/C18-clay (0–7 wt%) versus angular frequency; Figure S4: Master curves of (a) HTPB28/C18-clay (5–16 wt%) and (b) PB/C18-clay-12 wt% versus angular frequency. Solid symbols represent G′ and hollow symbols represent G″; Figure S5: Shift factors of the nanocomposites with different clay content (a) 0–7 wt%; (b) 5–16 wt%; Figure S6: Shear rate dependence of HTPB28/C18-clay-8 wt% at 20 °C; Figure S7: SAOS curves of HTPB28/C18-clay-8 wt% in different states – initial state (black), after LAOS shear (red), and after recovering for 5h from LAOS (blue). LAOS was applied with γ = 1.5 and ω = 10 rad/s at a temperature of T = 20 °C; Figure S8: (a)A homemade Teflon coaxial-cylinder fixture in 10 mm NMR glass tube was used to mimic shearing in rheometer (b)With the increase of pulse interval time, the signal in the MSE-FID decreased. In this paper, τ was set as 0.02 ms in the MAPE-DQ experiments; Figure S9: DQ intensity (normalized to the FID intensity by a single-pulse experiment) as a function of the DQ excitation time; Figure S10: (a) nDQ signal intensity as a function of DQ excitation time for the HTPB28/C18-clay-8 wt% nanocomposite in different states. (The solid lines were numeric fitting results via eq 6 in Supplementary Materials); (b) Dres distribution curves obtained from the numeric fitting on the nDQ curves in Figure S10a. All DQ experiments were performed at 30 °C.
