# Supplementary material for: Probing the Dynamic Structural Evolution of End-Functionalized Polybutadiene/Organo-Clay Nanocomposite Gels before and after Yielding by Nonlinear Rheology and 1H Double-Quantum NMR
Source: Polymers (Basel). 2022 Apr 8;14(8):1518. doi: 10.3390/polym14081518 (PMC9031593; doi:10.3390/polym14081518)
Supplement: Supplementary file 1 [file polymers-14-01518-s001.zip › polymers-1669972-supplementary.pdf]

polymers

MDPI

Supplementary Materials

Probing the Dynamic Structural Evolution of End-Functionalized Polybutadiene/Organo-Clay Nanocomposite Gels before and after Yielding by Nonlinear Rheology and ^{1}H Double-Quantum NMR

Wansu Peng [1], Chengdong Feng [1], Jiawen Hou [2], Rongchun Zhang [3], Pingchuan Sun [2], Yun Gao [1] and Xiaoliang Wang [1,*]

[1] Key Laboratory of High Performance Polymer Materials and Technology of Ministry of Education, Department of Polymer Science and Engineering, School of Chemistry and Chemical Engineering, Nanjing University, Nanjing 210023, China; mg1924065@smail.nju.edu.cn (W.P.); mg1924018@smail.nju.edu.cn (C.F.); p709@nju.edu.cn (Y.G.)

[2] Key Laboratory of Functional Polymer Materials of Ministry of Education and College of Chemistry and School of Physics, Nankai University, Tianjin 300071, China; 2120200839@mail.nankai.edu.cn (J.H.); spclbh@nankai.edu.cn (P.S.)

[3] South China Advanced Institute for Soft Matter Science and Technology (AISMST), School of Emergent Soft Matter, Guangdong Provincial Key Laboratory of Functional and Intelligent Hybrid Materials and Devices, South China University of Technology, Guangzhou 510640, China; zhangcr@scut.edu.cn

* Correspondence: wangxiaoliang@nju.edu.cn

Contents:

1. Transmission Electron Microscopy (TEM)

2. X-ray Diffraction (XRD)

3. Rheology Experiments.

4. Proton DQ Spectroscopy

1. Transmission Electron Microscopy (TEM).

TEM was carried out on a JEM 2100 electron microscope operated at 200 kV.

The TEM image of 8 wt% HTPB28/C18-clay nanocomposite, shown in Figure S1a, suggested that the organo-clay sheets were randomly dispersed in the polymer matrix after in-situ annealing on the rheometer. While in 16 wt% HTPB28/C18-clay nanocomposite, shown in Figure S1b, most of the organo-clay sheets were intercalated in the polymer matrix after in-situ annealing on the rheometer.

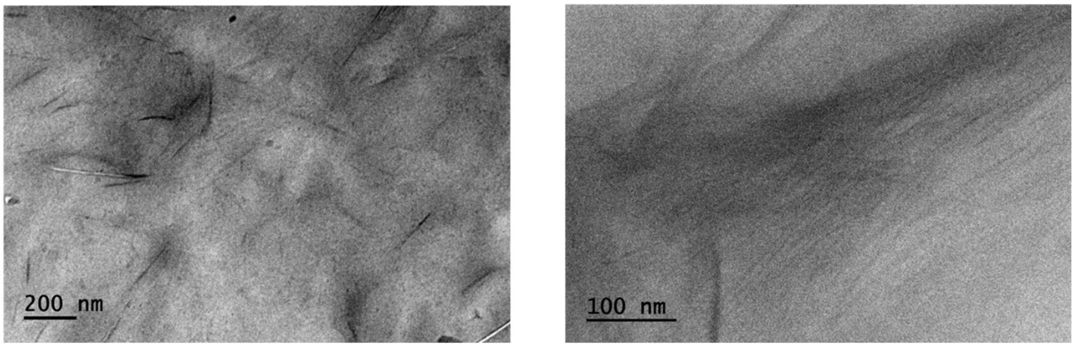

(**a**) (**b**)

Figure S1. TEM images of the (**a**) 8 wt% and (**b**) 16 wt% HTPB28/C18-clay nanocomposites after annealing above 120 °C.

2. X-ray Diffraction (XRD)

The microstructures of these nanocomposites have been determined by X-ray diffraction (Shimadzu XRD-6000 X-ray diffractometer with Cu Kα, λ = 0.154 nm, radiation at a generator voltage of 40 kV and a current of 30 mA).

As shown in Figure S2, the peak of 2θ at around 4° was a (001) basal reflection peak ascribed to stacked organo-clay sheets, which appeared in pure C18-clay and PB/C18-clay 8wt%. The peak at 4° disappeared in HTPB/C18-clay 8wt%, was not due to the low quantity of clay in the nanocomposite gels, as the (060) in-plane reflection peak of the single clay sheet could still be detected (the inset of Figure S2). This suggested a well exfoliated structure in HTPB/C18-clay 8wt%. The peak appeared again when more C18-clay was added, e.g. larger than 12 wt%, suggested the appearance of intercalated structures. The XRD results agreed well with TEM results in Figure S1.

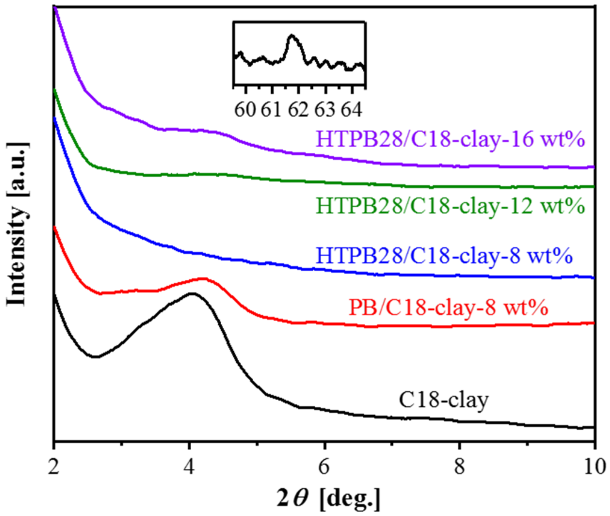

Figure S2. XRD patterns of the C18-clay, PB/C18-clay-8 wt%, HTPB28/C18-clay-8 wt%, HTPB28/C18-clay-12 wt% and HTPB28/C18-clay-16 wt%.

3. Rheology Experiments:

Rheology experiments were all carried out on a strain-controlled rheometer ARES-G2 (TA Instruments). A 25mm diameter parallel plate with the gap between 0.7-1.0 mm were used in linear viscoelastic measurements. Small amplitude oscillatory shear (SAOS) from 100 to 0.1 rad/s was performed in a temperature range from -60 to 20 °C (0-8 wt%)

and 0 to 120 °C (5-16 wt%) with an interval of 20 °C in the linear region (e.g. 1% strain rate). Master curves were obtained by time temperature superposition. The reference temperature (T_{ref}) was set at 20 °C.

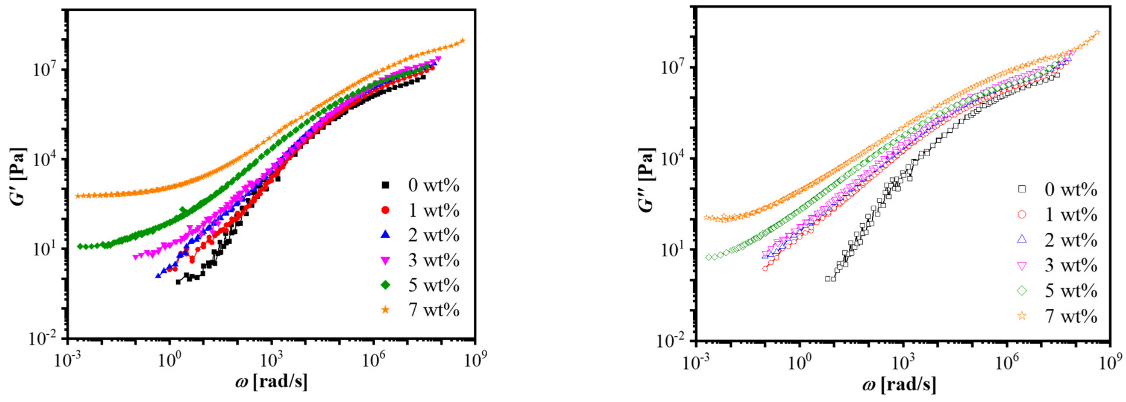

(**a**) (**b**)

Figure S3. Master curves of (**a**) *G′* and (**b**) *G′′* of low-concentration HTPB28/C18-clay (0–7 wt%) versus angular frequency.

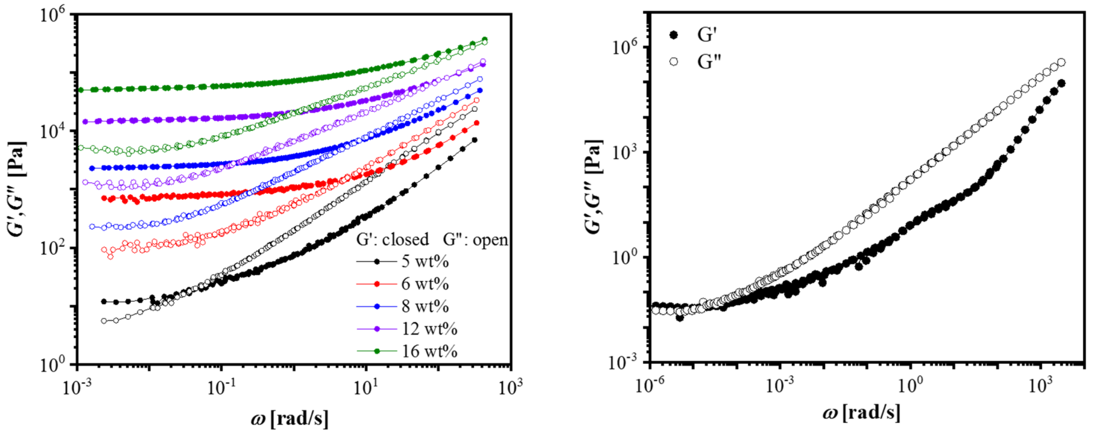

(**a**) (**b**)

Figure S4. Master curves of (**a**) HTPB28/C18-clay (5-16 wt%) and (**b**) PB/C18-clay-12 wt% versus angular frequency. Solid symbols represent *G′* and hollow symbols represent *G′′*.

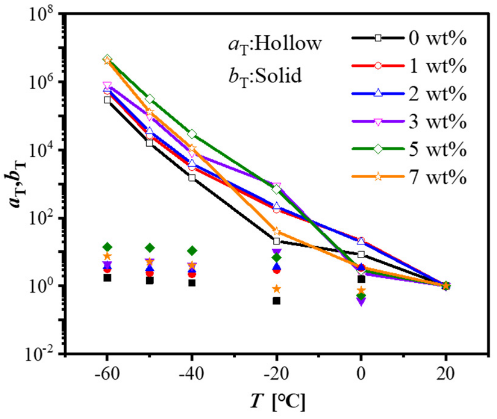

(a)

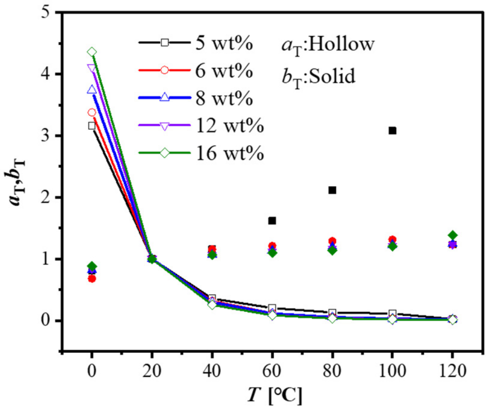

(b)

Figure S5. Shift factors of the nanocomposites with different clay content (a) 0-7 wt%; (b) 5-16 wt%.

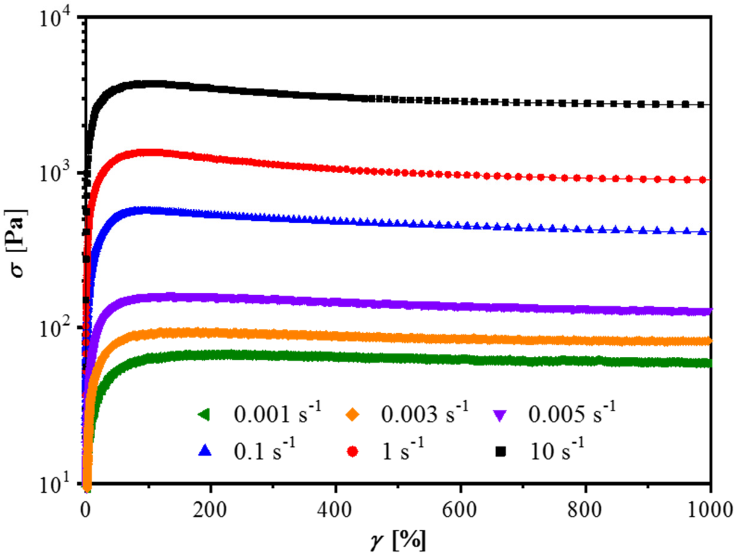

Figure S6. Shear rate dependence of HTPB28/C18-clay-8 wt% at 20 °C.

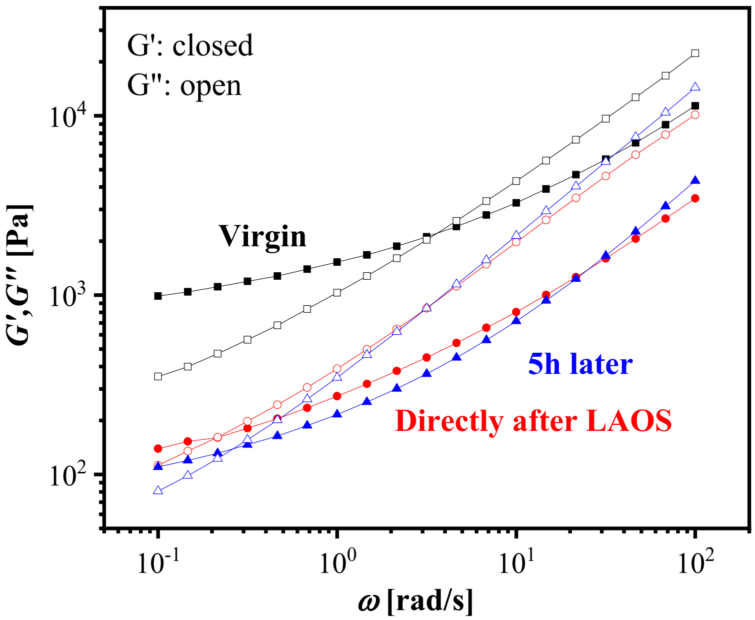

Figure S7. SAOS curves of HTPB28/C18-clay-8 wt% in different states–initial state (black), after LAOS shear (red), and after recovering for 5h from LAOS (blue). LAOS was applied with γ = 1.5 and ω = 10 rad/s at a temperature of T = 20 °C.

4. Proton DQ Spectroscopy

All the proton DQ NMR experiments were carried out on a Bruker Minispec mq20 at a proton resonance frequency of 20 MHz at 30 °C. The sample temperature was controlled by a BVT3000 heater with a accuracy of ± 0.1 °C. A homemade Teflon coaxial-cylinder (Figure S8a), i.e. a circular column with a diameter of 4 mm and height of 20 mm at the bottom of the column with diameter of 8 mm, was put into the 10 mm NMR tube, which could be used to yield the sample as we did in rheometer. The 90° pulse length is about 3.0 μs, and the receiver dead time is about 13 μs. MAPE-DQ experiment [1] were performed for all the samples, where a MAPE (magic and polarization echo) [2] dipolar filter was implemented right before the DQ recoupling sequences (i.e. Baum-Pine pulse sequence [3]) in order to eliminate the interference of the signals of rigid polymers closely attached to the nanosheets. The effects of different τ values (i.e. pulse delay between consecutive 90º pulses) were shown in Figure S8b. The finial τ was chosen as 0.02 ms, which was a tradeoff between the efficiency of filtering out the signals of rigid polymers attached on clay sheets and the signal intensity. Then the corresponding MAPE filter time was 0.32 ms (16 times τ).

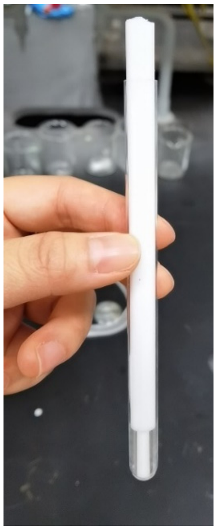

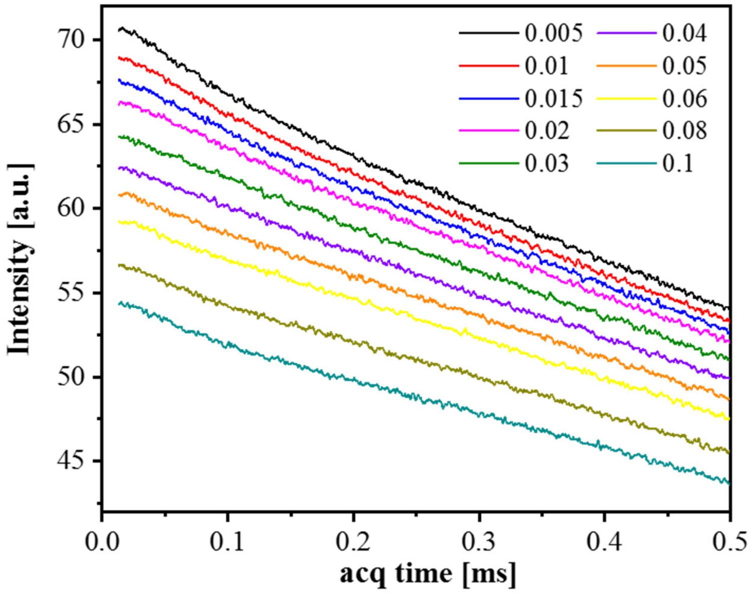

(**a**) (**b**)

Figure S8. (**a**)A homemade Teflon coaxial-cylinder fixture in 10 mm NMR glass tube was used to mimic shearing in rheometer (**b**)With the increase of pulse interval time, the signal in the MSE-FID decreased. In this paper, τ was set as 0.02 ms in the MAPE-DQ experiments.

From the DQ experiments, we could get two sets of data: I_{DQ} and I_{ref}, which are DQ and reference signal intensity, respectively. The buildup curve of I_{DQ} along with τ_{DQ}, as shown in Figure S9, suggested that the DQ signal intensity was proportional to the clay concentration due to the confinement effect of clays.

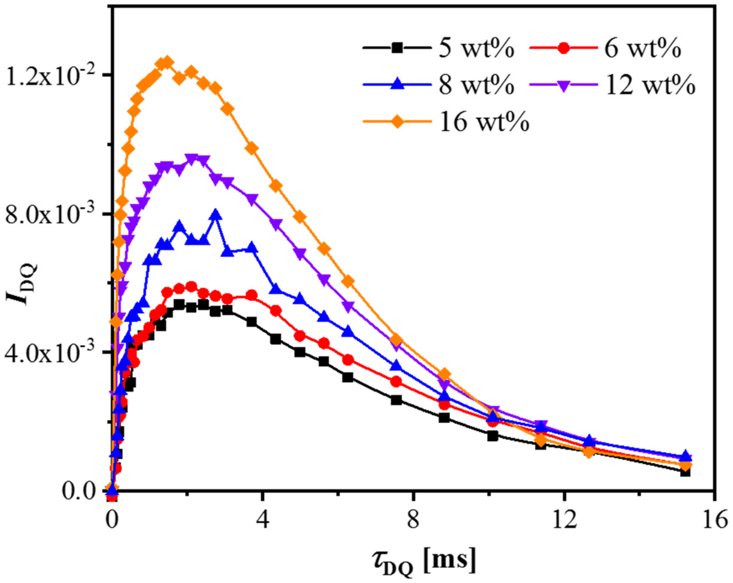

Figure S9. DQ intensity (normalized to the FID intensity by a single-pulse experiment) as a function of the DQ excitation time.

Furthermore, normalized DQ intensity could be used to quantitatively analyze the polymer network structure [4]:

$$I_{nDQ} = \frac{I_{DQ}}{I_{DQ} + I_{ref} - f_B e^{-2\tau_{DQ}/T_{2B}} - f_C e^{-2\tau_{DQ}/T_{2C}}} \quad (1)$$

f_B and f_C are the fractions of potential two separable isotropic mobile components with an apparent spin–spin relaxation time of T_{2B} and T_{2C}, respectively. By fitting the tail of the $I_{ref} - I_{DQ}$ curve due to their apparently slow relaxation behaviors [5], the obtained normalized I_{DQ} can be fitted through an Abragam-like kernel function [6]

$$I_{DQ}(\tau_{DQ}, D_{res}) = 0.5[1 - exp\{-(0.378 D_{res}\tau_{DQ})^{1.5}\} \times cos(0.583 D_{res}\tau_{DQ}) \quad (2)$$

by further assuming that D_{res} follows the log-normal distribution function P(D_{res}) [7]

$$I_{nDQ}(\tau_{DQ}) = \int P(D_{res}) I_{DQ}(\tau_{DQ}, D_{res}) dD_{res} \quad (3)$$

where

$$P(D_{res}) = \frac{1}{\sqrt{2\pi}\sigma D_{res}} e^{-(ln(D_{res}/D_m))^2/2\sigma^2} \quad (4)$$

On a logarithmic scale, P(D_{res}) can also be written as

$$P(ln\, D_{res}) = \frac{1}{\sqrt{2\pi}\sigma} e^{-(ln(D_{res}/D_m))^2/2\sigma^2} \quad (5)$$

Thus

$$\begin{aligned} I_{nDQ}(\tau_{DQ}) &= \int P(D_{res}) I_{DQ}(\tau_{DQ}, D_{res}) dD_{res} \\ &= \int \frac{1}{\sqrt{2\pi}\sigma D_{res}} e^{-(ln(\frac{D_{res}}{D_m}))^2/2\sigma^2} I_{DQ}(\tau_{DQ}, D_{res}) dD_{res} \\ &= \int \frac{1}{\sqrt{2\pi}\sigma} e^{-(ln(\frac{D_{res}}{D_m}))^2/2\sigma^2} I_{DQ}(\tau_{DQ}, D_{res}) d\, ln\, D_{res} \\ &= \int P(ln\, D_{res}) I_{DQ}(\tau_{DQ}, D_{res}) d\, ln\, D_{res} \end{aligned} \quad (6)$$

Here, σ is the standard deviation and is non-dimentional, indicating the inhomogeneity of D_{res} distribution and roughly corresponding to the full width at half-maximum (FWHM) of the D_{res} distribution curve on a logarithmic scale (eq 6). D_m is the median value for the D_{res} distribution, which reflectsthe residual dipolar couplings with maximum probability. Hence, it is applied as a good representative of the averaged dipolar couplings for the measured sample.

By applying several times of shearing on the initial annealed sample, the values of residual dipolar coupling and its distribution leveled off, as shown in Figure S10. This meant the yielded network structure changed no more by further manual shearing.

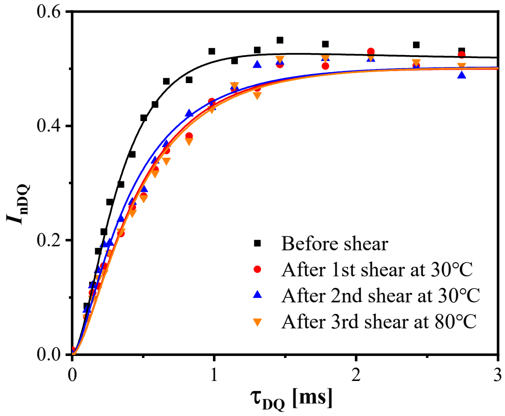

(a)

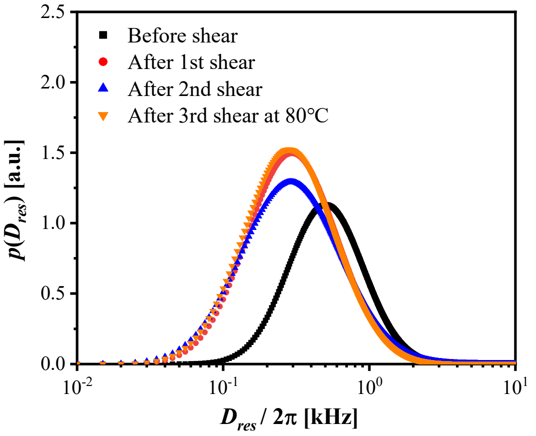

(b)

Figure S10. (**a**) nDQ signal intensity as a function of DQ excitation time for the HTPB28/C18-clay-8 wt% nanocomposite in different states. (The solid lines were numeric fitting results via eq 6 in Supplementary Materials); (**b**) D_{res} distribution curves obtained from the numeric fitting on the nDQ curves in Figure S10a. All DQ experiments were performed at 30 °C.

References

1. Zhang, R.C.; Yu, S.; Chen, S.L.; Wu, Q.; Chen, T.H.; Sun, P.C.; Li, B.H.; Ding, D.T. Reversible Cross-Linking, Microdomain Structure, and Heterogeneous Dynamics in Thermally Reversible Cross-Linked Polyurethane as Revealed by Solid-State NMR. J. Phys. Chem. B **2014**, *118*, 1126–1137, doi:10.1021/jp409893f.
2. Demco, D.E.; Johansson, A.; Tegenfeldt, J. Proton Spin-Diffusion for Spatial Heterogeneity and Morphology Investigations of Polymers. Solid State Nucl. Magn. Reson. **1995**, *4*, 13–38, doi:10.1016/0926-2040(94)00036-c.
3. Baum, J.P., A. . NMR Studies of Clustering in Solids. J. Am.Chem. Soc. **1986**, *108*, 7447–7454, doi.org/10.1021/ja00284a001.
4. Saalwachter, K. Proton Multiple-Quantum NMR for The Study of Chain Dynamics and Structural Constraints in Polymeric Soft Materials. Prog. Nucl. Magn. Reson. Spectrosc. **2007**, *51*, 1–35, doi:10.1016/j.pnmrs.2007.01.001.
5. Chasse, W.; Lang, M.; Sommer, J.U.; Saalwachter, K. Cross-Link Density Estimation of PDMS Networks with Precise Consideration of Networks Defects. Macromolecules **2012**, *45*, 899–912, doi:10.1021/ma202030z.
6. Chasse, W.; Valentin, J.L.; Genesky, G.D.; Cohen, C.; Saalwachter, K. Precise Dipolar Coupling Constant Distribution Analysis in Proton Multiple-Quantum NMR of Elastomers. J. Chem. Phys. **2011**, *134*, 10, doi:10.1063/1.3534856.
7. Lorthioir, C.; Randriamahefa, S.; Deloche, B. Some Aspects of The Orientational Order Distribution of Flexible Chains in A Diblock Mesophase. J. Chem. Phys. **2013**, *139*, 9, doi:10.1063/1.4838375.
